# Joint Expedition: Exploring Clinical Medical Imaging and Artificial Intelligence as a Team Integration

**DOI:** 10.3390/diagnostics14060584

**Published:** 2024-03-10

**Authors:** Daniele Giansanti

**Affiliations:** Centro Nazionale Tecnologie Innovative in Sanità Pubblica, Istituto Superiore di Sanità, Via Regina Elena 299, 00161 Roma, Italy; daniele.giansanti@iss.it

## 1. The Joint Expedition Exploring Clinical Medical Imaging and Artificial Intelligence

The field of clinical medical imaging has seen remarkable advancements in recent years, particularly with the introduction of artificial intelligence (AI) techniques. AI has the potential to revolutionize clinical medical imaging by enabling more accurate, efficient, and personalized diagnoses and treatments.

The landscape of clinical imaging is currently undergoing a revolutionary metamorphosis (contribution 1), with every sector impacted by the integration of artificial intelligence (AI). This paradigm shift is not confined to one specific domain but spans the diverse realms of medical imaging, encompassing imaging diagnostics for organs and functionality [1,2], the dynamic field of digital pathology (encompassing both cytology and digital histology) [3,4], the intricacies of digital dermatology [5,6], and various other niches within the expansive field of clinical imaging.

The infusion of AI into these sectors is not merely a superficial addition but a fundamental reshaping of their technological fabric. The integration processes, fuelled by advancements in AI technologies, experienced a noteworthy acceleration, with the upheavals brought about by the COVID-19 pandemic further propelling this momentum [7,8].

In the face of these transformative developments, it becomes increasingly crucial to direct attention not only towards the driving force of scientific and technological innovation instigated by AI but also towards the intricate process of embedding AI seamlessly into the broader health domain. This integration is pivotal not just for the sake of technological advancement but as a strategic imperative to optimize healthcare practices, enhance diagnostic accuracy, streamline workflows, and ultimately elevate the capabilities of clinical imaging to new heights within the dynamic landscape of modern medical science.

In light of these considerations, we introduced a Special Issue entitled Artificial Intelligence in Clinical Medical Imaging: https://www.mdpi.com/journal/diagnostics/special_issues/3FXN9682V0, accessed on 29 February 2024.

The objective was to comprehensively outline the ongoing developments, share established experiences, explore prospects, and highlight persisting challenges in this dynamic field.

The Special Issue successfully achieved a significant milestone, featuring 13 contributions (Co)s (excluding this editorial) (Co. 1–Co. 13).

The published papers, according to the selected categories, encompass 1 introductory editorial (Co. 1), 10 full scientific articles (Co. 2–Co. 11), 1 review (Co. 12), and 1 comment (Co. 13).

## 2. Conclusive Discoveries: A Closer Look at the Contributions

### 2.1. An Overview of the Contributions

Below, we present a concise overview encapsulating the key points and insights from the contributions featured in the special issue. This summary aims to provide a brief yet comprehensive glimpse into the diverse and impactful content published within this specialized collection.

#### 2.1.1. Pirrera, A. et al. (Co. 1): Exploring the Synergy in Clinical Imaging with Artificial Intelligence

The Editorial by Pirrera and Giansanti (Co. 1) introduced the aims of the SI and reflected on the progress and status of the introduction of AI into clinical medical artificial intelligence. The focus was on assessing the current state and briefly exploring both the evolution and the recent trends. The editorial introduced the need for an initiative Special Issue and suggested fields and directions for exploration.

The 12 contributions (Co. 2–13) covered various topics of interest to the SI. Below are detailed the focus and a brief excerpt of the content.

#### 2.1.2. Lin, P.-C. et al. (Co. 2): Machine Learning for Lumbar Disc Height Correlation on X-rays

The study focuses on lumbar disc bulging or herniation (LDBH), a major cause of spinal issues necessitating surgery. Due to limited access to MRI, lumbar X-rays are explored for diagnostic support. Analyzing 458 patients, machine learning methods identify key predictors, with L4-5 posterior disc height, age, and L1-2 anterior disc height emerging as crucial factors. A decision tree algorithm is proposed as a valuable tool for clinical decision-making by surgeons. The study underscores the importance of machine learning-based decision tools, particularly highlighting the role of L1-2-disc height in the context of LDBH. Future research aims to develop a comprehensive decision-support model.

#### 2.1.3. Stanojević Pirković, M. et al. (Co. 3): Fractional Flow Reserve-Based Patient Risk Classification

The study addresses the global impact of cardiovascular diseases (CVDs), emphasizing the significance of preventing and detecting risks, particularly focusing on acute myocardial infarction (AMI) responsible for 3 million deaths annually. The research aims to develop a technique using fractional flow reserve (FFR) measurements for patient evaluation and predicting the risk of death. A random forest machine learning model is employed, achieving a 76.21% prediction accuracy, with mean accuracies ranging from 74.1% to 83.6% across different test sample sizes. Additionally, a numerical approach involving the 3D reconstruction of coronary arteries for stenosis monitoring is implemented, showing promising results even with limited data. The study suggests that future improvements can be achieved by incorporating additional data, enabling the exploration of different machine learning algorithms.

#### 2.1.4. Rao, P.K. et al. (Co. 4): Efficient Kidney Tumor Segmentation with UNet-PWP Deep-Learning Model on CT Scan Images

This study addresses the complexity of early detection in kidney tumors, introducing the UNet-PWP architecture tailored for efficient segmentation. Notably, adaptive partitioning breaks down the UNet architecture into smaller submodels, optimizing computational resources. The model incorporates pre-trained weights, boosting its capacity for intricate tasks, and employs weight pruning for further efficiency without compromising performance. The evaluation against the DeepLab V3+ model on the “KiTs 19, 21, and 23” kidney tumor dataset demonstrates the UNet-PWP model’s outstanding 97.01% accuracy on both training and test datasets, outperforming the DeepLab V3+ model. To enhance interpretability, the study fuses attention and Grad-CAM XAI methods, providing valuable insights into decision-making and critical regions of interest. This interpretability is crucial for healthcare professionals to trust and understand the model’s reasoning, making the UNet-PWP architecture a promising advancement in kidney tumor segmentation.

#### 2.1.5. Kaur, M. et al. (Co. 5): ESRNet for Efficient Brain Tumor Classification

This paper introduces an Efficient Skip Connections-Based Residual Network (ESRNet) to address challenges in brain tumor classification using deep learning. ESRNet utilizes ResNet with skip connections to overcome limitations like vanishing gradient issues. It employs multiple stages with increasing residual blocks for enhanced feature learning and pattern recognition. The architecture ensures smooth gradient flow during training, preventing information loss. ESRNet integrates downsampling techniques and batch normalization for robust performance. Experimental results demonstrate ESRNet’s superior accuracy, sensitivity, specificity, F-score, and Kappa statistics, with median values of 99.62%, 99.68%, 99.89%, 99.47%, and 99.42%, respectively. The proposed ESRNet showcases exceptional efficiency in brain tumor classification, offering potential advancements in clinical diagnosis and treatment planning.

#### 2.1.6. Chen, Y.-Y. et al. (Co. 6): Bone Metastases Segmentation on Breast Cancer Bone Scans

This study focuses on employing deep learning for the automatic detection and quantification of bone metastases in bone scan images, providing clinical assistance in diagnosis. Using an internal dataset of breast and prostate cancer patients, the study adopts the Double U-Net model with modifications for multi-class segmentation. Techniques like Otsu thresholding, negative mining, background pre-processing, and transfer learning are employed to enhance model performance. Through 10-fold cross-validation, the best model achieves a precision of 69.96%, a sensitivity of 63.55%, and an F1-score of 66.60%. Compared to the baseline model, this represents improvements of 8.40%, 0.56%, and 4.33%, respectively. The developed system holds the potential for providing pre-diagnostic reports to aid physicians in final decisions and calculating the bone scan index (BSI) when combined with bone skeleton segmentation.

#### 2.1.7. Wu, H. et al. (Co. 7): One-Stage Detection for Multi-Type Coronary Lesions with Deep Learning

This study introduces a rare approach using a one-stage model, YOLOv5, for the automatic detection of coronary lesions without segmentation. Enrolling 200 patients with significant coronary issues, the images were categorized into two views. YOLOv5 demonstrated precision, recall, mAP@0.1, and mAP@0.5 at the image level, with values ranging from 0.66 to 0.73. At the patient level, the model exhibited precision, recall, and F1 scores, ranging from 0.64 to 0.65 and 0.91 to 0.94. YOLOv5 performed best for Chronic Total Occlusion (CTO) and Local Stenosis (LS) lesions. The study concludes that YOLOv5 is feasible for automatic coronary lesion detection, particularly for LS and CTO types.

#### 2.1.8. Jönemo, J. et al. (Co. 8): Augmentation Methods for Autism Classification with 3D CNN

This study explores the application of deep learning to resting-state functional MRI data for classifying subjects as healthy or having autism spectrum disorder. Notably, the focus is on investigating the impact of various 3D augmentation techniques on test accuracy. Using derivatives from 1112 subjects in the ABIDE dataset, a 3D Convolutional Neural Network (CNN) is trained. The findings reveal that while augmentation is employed, it only leads to minor improvements in test accuracy. This highlights the limited impact of 3D augmentation in enhancing classification performance in the context of neuroimaging data.

#### 2.1.9. Bhimavarapu, U. et al. (Co. 9): Automatic Diabetic Retinopathy Detection with CNN

This study focuses on the diagnosis of Diabetic Retinopathy (DR), a diabetes-associated eye disease with the potential for blindness. Employing deep learning for automatic DR diagnosis from fundus images, the study introduces an enhanced Convolutional Neural Network (CNN) model. The improved model incorporates a novel pooling function within the ResNet-50 architecture, aiming to increase diagnostic accuracy while reducing computational complexity and processing time. Trained and tested on APTOS and Kaggle datasets, the proposed model achieves impressive accuracies of 98.32% and 98.71%, respectively. The comparative analysis highlights the superior performance of the proposed model in DR diagnosis when compared to state-of-the-art approaches with retinal fundus images.

#### 2.1.10. Wu, S. et al. (Co. 10): Coarse-to-Fine Fusion Network for Small Liver Tumor Detection

This paper presents a novel approach for liver tumor semantic segmentation in medical image analysis, focusing on small tumors across various sizes. The proposed method integrates a detection module and a CSR (convolution-SE-residual) module, featuring a convolution block, an SE module, and a residual module for fine segmentation. Evaluating a private liver MRI dataset with 3605 tumors, including 3273 smaller than 3.0 cm, the method outperforms single-stage end-to-end networks and fusion networks, demonstrating superiority over 3D UNet and nnU-Net. In testing on 44 images, the proposed method achieves an average Dice similarity coefficient (DSC) and recall of 86.9% and 86.7%, respectively, surpassing comparison methods. Notably, for small objects (<10 mm), the proposed approach sets a state-of-the-art performance with a Dice score of 85.3% and a malignancy detection rate of 87.5%.

#### 2.1.11. Bragança, C.P. et al. (Co. 11): Advancements in AI for Glaucoma Diagnosis

This article delves into the increasing role of artificial intelligence (AI) algorithms in digital image processing and the automated diagnosis of glaucoma, a significant eye disease. It provides an overview of glaucoma types, traditional diagnostic methods, and the global epidemiology of the disease. The focus is on how AI algorithms can potentially aid in early glaucoma diagnosis through population screening. The related work section explores key studies and methodologies utilizing AI for the automatic classification of glaucoma from digital fundus images. It also highlights the main databases with labeled glaucoma images available for training machine learning algorithms.

#### 2.1.12. Giansanti, D. (Co. 12): Umbrella Review of fMRI and AI Fusion in Autism

This study conducts an umbrella review analyzing emerging themes in the integration of Functional Magnetic Resonance Imaging (fMRI) and artificial intelligence (AI) in autism diagnosis. Utilizing a structured process, it reviews 20 systematic reviews, emphasizing the significance of technological integration, especially fMRI and AI. The study acknowledges the potential in this field while recognizing challenges and limitations. It notes a growing emphasis on AI research but highlights the need for attention to healthcare process integration, including regulation, acceptance, informed consent, and data security. The study suggests focusing on health domain integration for routine implementation of these applications, pointing out the promising yet unexplored area of integration into personalized medicine (PM) in autism research.

#### 2.1.13. Giansanti, D. (Co. 13): AI-Enabled Fusion for Enhanced Autism Spectrum Disorder Diagnosis

The proposal is a comment on contribution 8. It underscores the substantial enhancement achieved in the realms of diagnosis and classification through the incorporation of 3D augmentation. This augmentation, when synergistically combined with artificial intelligence (AI) and Functional Magnetic Resonance Imaging (fMRI), presents a formidable approach. The integration of these technologies not only elevates the accuracy and efficacy of diagnostic processes but also holds the potential to unravel more nuanced insights into the intricacies of the data, thereby further refining our understanding and application of advanced medical imaging techniques.

### 2.2. Conclusive Global Reflection

All the works have made noteworthy contributions to the field of clinical medical imaging, particularly at the intersection with AI. These contributions provide valuable insights and innovative approaches, enhancing our understanding of how AI can improve medical imaging processes. The integration of artificial intelligence highlighted in these studies has practical implications for advancing diagnostic accuracy, efficiency, and overall capabilities in the realm of clinical medical imaging, signifying a significant step forward in this domain.

## 3. Common Message, Key Emerging Themes, and Suggestions for a Broader Investigation

### 3.1. Common Messages

Twelve distinct contributions (Co. 2–13) weave through the intricate field of medical research focused on the integration of clinical medical imaging with AI. These studies, spanning diverse medical domains, collectively leverage several AI approaches, making an important contribution to the landscape of medical diagnostics.

Commencing with lumbar disc exploration (Co. 2), the ensemble progresses into the cardiovascular sector (Co. 3), where a novel technique employing fractional flow reserve measurements proves adept at predicting patient risks. Kidney tumor detection takes a prominent role in Co. 4, introducing an architecture optimizing computational resources with impressive accuracy. In the cerebral domain of brain tumor classification (Co. 5), ESRNet showcases notable accuracy. Transitioning to bone metastases segmentation (Co. 6), a Double U-Net model enhances precision and sensitivity. A revolutionary note emerges in Co. 7, where a one-stage model (YOLOv5) redefines coronary lesion detection, excelling in specific lesion types. Co. 8 delves into autism classification, shedding light on the nuanced effectiveness of 3D augmentation techniques.

Ocular health comes to the fore both in Co. 9 and Co. 11. Co. 9 presents an improved ResNet-50 model for Diabetic Retinopathy diagnosis with remarkable accuracy, while Co. 11 investigates the application of AI in glaucoma diagnosis. Co. 10 introduces a novel semantic segmentation approach for liver tumors, outperforming existing models, particularly in segmenting small objects. Co. 12, an umbrella review, delves into fMRI and AI integration for autism diagnosis, recognizing potential and addressing challenges.

The last contribution, Co. 13 provides a perspective, underscoring the enhancement achieved through 3D augmentation, AI, and fMRI integration. This fusion refines diagnostic accuracy and unravels nuanced insights into advanced clinical medical imaging techniques, marking a moment in the evolution of medical diagnostics.

### 3.2. Suggestions for a Broader Investigation and Key Emerging Themes

From the overview, it is also possible to detect the emerging themes and the suggestions for a broader investigation.

The collective contributions (Co. 2–13) encompass diverse facets of medical imaging, showcasing the evolving landscape of artificial intelligence in healthcare. Lin et al. (Co. 2) illuminate lumbar disc issues through machine learning, prompting collaborative endeavors across orthopedics and technology. Stanojević Pirković’s work (Co. 3) on cardiovascular diseases encourages merging datasets for a holistic approach. Rao et al. (Co. 4) advocate for transparent kidney tumor segmentation models, emphasizing interpretability to instill trust. Efficient Brain Tumor Classification (Co. 5) prompts exploration of real-world applications, while ESRNet (Co. 6) advocates personalized medicine integration.

Chen et al. (Co. 7) propose one-stage models for coronary lesion detection, urging longitudinal studies. Jönemo’s insights (Co. 8) on autism classification with 3D CNNs suggest collaborative human–AI analysis. Bhimavarapu’s study (Co. 9) emphasizes scalable AI models for Diabetic Retinopathy screening. Ethical considerations in liver tumor detection (Co. 10) underscore the need for responsible AI use. Glaucoma diagnosis (Co. 11) pushes for patient-centric AI integration, acknowledging diverse perspectives. The Umbrella Review (Co. 12) highlights the potential of fusing fMRI and AI in autism with calls for ethical healthcare integration. The comment (Co. 13) applauds the synergy of 3D augmentation, AI, and fMRI, envisioning nuanced insights into medical imaging’s intricacies.

Through this experience, we identified noteworthy dominant themes, which are detailed in Table 1, along with the reference contributions. These discerned themes can serve as valuable inspiration for fellow researchers delving into this field.

## 4. Conclusions

In conclusion, the evolution of artificial intelligence technologies in the field of medical imaging offers promising prospects for enhancing diagnosis and treatment. The studies presented in this editorial highlight the growing intersection between medicine and artificial intelligence, addressing challenges from early spinal pathology diagnosis to Efficient Brain Tumor Classification. The research emphasizes the crucial aspects of interpretability and encourages multidisciplinary collaboration, providing valuable insights for further ethical investigations and practical applications.

The Special Issue curated significant contributions in various domains, identifying both emerging and established themes and delineating intriguing directions for future advancements. This initiative underscores the importance of these tools as a central hub for scholarly exchange and discussions among researchers worldwide.

## Figures and Tables

**Table 1:** Dominant emerging theme by article.

Themes	Description	Studies
Spinal and Skeletal Insights	Machine Learning for Lumbar Disc Height Correlation on X-rays. Bone Metastases Segmentation on Breast Cancer Bone Scans.	(Co. 2) (Co. 6)
Cardiovascular Precision	Fractional Flow Reserve-Based Patient Risk Classification. One-Stage Detection for Multi-Type Coronary Lesions with Deep Learning.	(Co. 3) (Co. 7)
Renal and Hepatic tumor detection/segmentation	Efficient Kidney Tumor Segmentation with UNet-PWP Deep-Learning Model on CT Scan Images. Coarse-to-Fine Fusion Network for Small Liver Tumor Detection.	(Co. 4) (Co.10)
Neurological Exploration	ESRNet for Efficient Brain Tumor Classification. Augmentation Methods for Autism Classification with 3D CNN. AI-Enabled Fusion for Enhanced Autism Spectrum Disorder Diagnosis.	(Co. 5) (Co. 8) (Co. 13)
Ocular Health Focus	Co. 9: Automatic Diabetic Retinopathy Detection with CNN. Co. 11: Advancements in AI for Glaucoma Diagnosis	(Co. 9) (Co. 11)
AI and fMRI	Umbrella Review of fMRI and AI Fusion in Autism	(Co. 12)
